# Video game addiction among Tunisian adolescents in Sousse: A cross-sectional study in high schools

**DOI:** 10.1093/eurpub/ckac129.520

**Published:** 2022-10-25

**Authors:** M Ouertani, S Ben Fredj, R Ghammem, N Zammit, A Maatouk, W Ben Belgacem, S Boujebha, N Guesmi, J Maatoug, H Ghannem

**Affiliations:** 1 Epidemiology Department, Farhat Hached Hospital, Sousse, Tunisia; 2 Faculty of Medicine of Sousse, University of Sousse, Sousse, Tunisia

## Abstract

**Background:**

Addiction to video games is a theme regularly mentioned and associated with the risks that concern adolescents. This study aimed to determine the prevalence of video game addiction (VGA) among adolescents and identify its associated factors.

**Methods:**

We conducted a cross-sectional study within public high schools, in Sousse, Tunisia in 2019. The target population was high school students. A structured self-administered questionnaire was used to collect data about sociodemographic characteristics, lifestyle behaviors, and mental health disorders, and we used the 21-item Game Addiction Scale to objectify video game addiction. Statistical analysis was carried out using the program SPSS v.20.

**Results:**

A total of 1342 participants were recruited for the study,36.8% of whom were boys. The average age was 17.5 ±1.44 years. The analysis of mental health disorders showed that 67% were anxious, 66.8% were alexithymic, 65.4% were depressed and 39% had low self-esteem. The analysis of lifestyle behaviors showed that a lack of physical activity was reported by 57.6% of participants. Problematic use of the Internet (>2 hours per day) was reported among 72.4% of the students. The prevalence of video game addiction was 13%. Boys were more prone to be addicted than girls (66.2% versus 33.8%, p < 0.001). ≥17 years old students had a higher rate of VGA than those aged<17 years old (57.3% vs 42.7%, p = 0.031). VGA was higher among students who follow the non-scientific study section (69.4%, p = 0.007). Students who had moderate depression had the highest percentages of VGA (35.7%, p = 0.005). Students who had problematic use of the internet were more addicted to video games (80.3%) than those not having problematic use (19.7%), p = 0.009.

**Conclusions:**

This study allowed us to identify the students who are vulnerable to VGA. Also, a huge responsibility is accorded to school staff and parents to tackle this health problem by sensitizing their children and setting up some protective family rules.

**Key messages:**

• Several factors were significantly associated with video game addiction, especially problematic internet use.

• Further research is needed to understand the underlying mechanisms of video game addiction and to explore effective preventive or interventional strategies.

